# Comparison of the Effect of Glycemic Control in Type 2 Diabetes Outpatients Treated With Premixed and Basal Insulin Monotherapy in China

**DOI:** 10.3389/fendo.2018.00639

**Published:** 2018-10-29

**Authors:** Guangxu Liu, Jingtao Dou, Yuesong Pan, Yuxiang Yan, Huiping Zhu, Juming Lu, Herbert Gaisano, Linong Ji, Yan He

**Affiliations:** ^1^Department of Epidemiology and Biostatistics, School of Public Health, Capital Medical University, Beijing, China; ^2^Beijing Municipal Key Laboratory of Clinical Epidemiology, Beijing, China; ^3^Department of Endocrinology, Chinese PLA General Hospital, Beijing, China; ^4^Department of Endocrinology, Hainan Branch of Chinese PLA General Hospital, Sanya, China; ^5^Departments of Medicine and Physiology, University of Toronto, Toronto, ON, Canada; ^6^Department of Endocrinology and Metabolism, Peking University People's Hospital, Beijing, China

**Keywords:** premixed insulin, basal insulin, type 2 diabetes mellitus, hemoglobin A1c, chinese outpatients

## Abstract

**Background:** Basal and premixed insulin have been widely used for insulin therapy of type 2 diabetes mellitus (T2DM) in China. The aim of this study is to compare the sustained efficacy of basal and premixed insulin therapies in T2DM outpatients with insulin monotherapy.

**Materials and Methods:** The survey was conducted in 602 hospitals across China from April to June in 2013. The participants included outpatients who were receiving basal or premixed insulin monotherapy for more than 3 months, and the outcome was attaining a glycated hemoglobin A1C (HbA1c) of <7.0% as a measure of sustained glycemic control.

**Results:** A total of 49,119 T2DM outpatients on basal (*n* = 11,967) or premixed insulin (*n* = 37,152) monotherapy were included in the final analyses. Using multivariable model analysis, patients using premixed insulin exhibited a better glycemic control, with more outpatients achieving the target HbA1c level than those using basal insulin (model 1, OR 0.695, 95%CI 0.664–0.728; model 2, OR 0.708, 95%CI 0.676–0.742; model 3, OR 0.717, 95%CI 0.684–0.752; model 4, OR 0.750, 95%CI 0.715–0.787). Using subgroup analysis stratified by age, sex, duration of diabetes, duration of insulin treatment, and complications, still more outpatients in every subgroup treated with premixed insulin achieved the target HbA1c (HbA1c < 7%) than those receiving basal insulin.

**Conclusions:** Premixed insulin monotherapy had a better glycemic control (HbA1c < 7.0%) than basal insulin monotherapy for Chinese T2DM outpatients in daily.

## Introduction

Diabetes mellitus is a worsening world-wide health problem, with an increasing prevalence, especially in Asian countries (1). A latest national survey reported that the prevalence of type 2 diabetes mellitus (T2DM) hit 10.9% in adult populations in China in 2013 (1). The International Diabetes Federation (IDF) estimated that China had 90 million diabetic patients in 2011, and that this number was expected to exceed 129.7 million by the year 2030 (2), which makes diabetes mellitus a most alarming public health problem in China.

The conventional management of T2DM include lifestyle measures of a healthy diet, regular exercise and some weight loss; and if these fail, then to start anti-diabetic agents at or soon after the diagnose and/or insulin therapy (3, 4). For the latter, there exists different types of insulin and insulin regimens, but there has not been an overall consensus regarding the most effective or optimal insulin regimen for patients with diabetes mellitus, after the failure of lifestyle measures and oral antidiabetic agents (OAD) to achieve a sustained glycemic control as determined by the glycated hemoglobin A1C (HbA1c) target of <7% (5–7). Among insulin regimens, basal and premixed insulin have been widely used for insulin therapy of type 2 diabetes mellitus (T2DM) (8–12). Basal insulin is a single dose intermediate- or long-acting insulin. Premixed insulin is a combination of two insulins mixed together, one that is short-acting or fast-acting, plus one that is intermediate-acting. Some clinical guidelines recommended a basal insulin regimen as the initiation insulin therapy for hyperglycemia in non-critically ill patients (10, 12), whereas a premixed insulin formulation is also a most frequently prescribed treatment for patients with T2DM in many regions in Asia, Europe, and Latin America (8, 9, 11).

In China, Ji and colleagues showed that after failure of oral hypoglycemic medications, initiating treatment with premixed insulin was able to achieve glycemic control in 70% of T2DM patients (13). A systematic review study of randomized controlled trials showed that premixed insulin can be a simple and effective means of T2DM treatment in East Asians, and its safety is generally similar to that of basal or basal-bolus insulin (14). In the hospital setting, premixed insulin was reported to be effective in improving hyperglycemia and controlling HbA1c in T2DM inpatients, and the effect was also similar to inpatients receiving basal insulin (15). In China, several clinical studies reported that premixed insulin was similar in efficacy and safety to basal insulin for T2DM inpatients (16, 17). To the best of our knowledge, no such study comparing basal and premixed insulin regimens has been performed in the T2DM outpatients with insulin monotherapy in China. This study used the data from the large-scale study of China National HbA1c Surveillance System (CNHSS), which was designed to monitor the HbA1c control and investigated the ways of treatment of T2DM patients, to compare the efficacy of basal and premixed insulin treatments for T2DM outpatients who were treated with insulin monotherapy.

## Materials and methods

### Study design and participants

In this analysis, we used the data from survey of CNHSS which was conducted by the Chinese Diabetes Association and mainly aimed at monitoring the HbA1c control and investigating the treatments of type 2 diabetes outpatients in China. Six hundred and two hospitals, including 13 primary care hospitals, 132 secondary care hospitals, and 457 tertiary care hospitals across the China mainland, were involved in this study from April to June, 2013, which basically covered almost all the provincial administrative regions, except the Tibet and Guangxi Zhuang autonomous regions. A primary hospital was a community medical institution and provided primary health services; a secondary hospital was a local medical institution and provided comprehensive health services; and a tertiary hospital was a regional medical institution and provided comprehensive and specialist health services. All involved provinces were divided into undeveloped region, intermediately developed region and developed region according to geographical distribution. All T2DM outpatients from the involved hospitals provided informed written consent before being entered into the study. Ethics approval was obtained from the Ethics Committee for Clinical Research of the Chinese People's Liberation Army General Hospital, and which was also accepted by all of the participating hospitals.

In each workday during the survey period, the first consecutive 7 outpatients who entered each hospital's Endocrinology outpatient department that met the eligibility criteria were invited to participate in the survey, until 400 patients were recruited from each involved hospital. The inclusion criteria were: (1) at least one previous outpatient medical record pertaining to T2DM according to WHO 1999 diagnostic criteria (18); (2) a T2DM outpatient treated with oral antidiabetic agents (OADs) alone, OADs combined with insulin, insulin monotherapy or OADs combined with glucagon-like peptide-1 (GLP-1) therapy; and (3) a local resident for at least 6 consecutive months with an age of 18 years or older; The exclusion criteria included: (1) diabetes secondary to other diseases; (2) inpatients; (3) pregnant or breast-feeding; (4) unable to complete the survey; and (5) unconsciousness or unable to communicate. In this survey, total of 238,639 outpatients with T2DM were recruited, which included 114,284 T2DM outpatients (47.9%) that were treated with only OADs, 60,105 (25.2%) outpatients received OADs combined with insulin or GLP-1 drugs, 453 outpatients (0.2%) accepted only a GLP-1 drug, 5546 (2.3%) with lifestyle therapy, and 58,251 (24.4%) outpatients were treated with insulin monotherapy. Among the outpatients treated with insulin monotherapy, 6,232 (10.7%) patients were treated with basal-bolus insulin, 2,310 (4.0%) with prandial insulin only, and wherein 590 (1.0%) was treated for <3 months and were therefore excluded. Finally, the majority of 49,119 outpatients (84.3%) who were treated with either basal insulin once daily (*n* = 11,967) or premixed insulin twice daily (*n* = 37,152) were included in the analysis for this study. The HbA1c of <7.0% was defined as reaching the goal of satisfactory glycemic control (19).

### Data collection

Demographic information, including age, gender, was collected using a short questionnaire by face-to-face interview with the endocrinologists, nurses or post-graduates from the involved hospitals. Clinical characteristics were obtained by checking the medical records, which included date of first diagnosis of diabetes, and medical history of hypertension, coronary heart disease, cerebrovascular disease, dyslipidemia, diabetic retinopathy, diabetic neuropathy, diabetic nephropathy and diabetic foot. Laboratory examination of fasting plasma glucose (FPG), 2-h postprandial plasma glucose (2h-PPG) and HbA1c and a physical examination which included body height and body weight before insulin therapy were also checked through the medical record by the interviewer. Body mass index (BMI) was calculated as body weight in kilograms divided by squared body height in meters. The HbA1c after insulin therapy was measured by standard centralized measurement by the involved hospitals. A designated researcher entered in all the data and uploaded the data to the central database of the CNHSS.

### Definition of insulin treatment

Basal insulin is a medium or long acting form of insulin. In this study basal insulin included an intermediate-acting insulin (neutral protamine Hagedorn, NPH) and the long-acting insulin (such as insulin glargine or insulin detemir) once-daily. Premixed insulin is a proportionate mixture of a short-acting or fast-acting insulin and an intermediate-acting insulin. In this study, premixed insulin preparations included Novolin Mix 30 (30% soluble insulin aspart injection and 70% protamine-crystallized insulin aspart, Novo Nordisk Pharmaceutical Company, China), Humalog Mix 25 or 50 (25 or 50% insulin lispro and 75 or 50% neutral protamine lispro, Eli Lilly and Company, USA), Novolog Mix 30R or 50R (30 or 50% insulin aspart injection and 70 or 50% insulin aspart protamine suspension, Novo Nordisk Pharmaceutical Company, China), Gansulin 30R or 40R (30 or 40% regular insulin and 70 or 60% NPH, Dongbao Pharmaceutical, China) and USLIN 30R or 50R (30 or 50% recombinant human insulin and 70 or 50% insulin protamine suspension, Unit Pharmaceutical, China).

### Statistical analysis

All analysis was performed by the Statistical Analysis System (SAS) release 9.4 (SAS Institute Inc., Cary, NC, USA). Continuous variables of clinical characteristics were presented as mean with standard deviation and categorical variables as frequency with proportions. Forest plot was created by R version 3.3.1 (R development core team; available from http://www.r-project.org/).

Chi-square test was used to compare categorical variables and *t*-test/Wilcoxon rank test was used to compare continuous variables between patients with different type of insulin therapy. Multiple logistic regression analysis was used to compare the effects of the different insulin regimens to achieve the target HbA1c of <7% in all patients or by subgroup analysis. All values of *P* < 0.05 for two-side tests were considered statistically significant.

## Results

### Clinical characteristics of participants prior to insulin therapy

Among the 49,119 T2DM outpatients who received insulin monotherapy in this study, 11,967 (24.4%) received basal insulin monotherapy, and 37,152 (75.6%) received premixed insulin monotherapy. Demographic information and clinical characteristics of the participants prior to insulin therapy are shown in Table 1. The characteristics between the two groups are not significantly different for gender (*P* = 0.277), hospital level (*P* = 0.940), and the proportion achieving target FPG (*P* = 0.064) and HbA1c (*P* = 0.054). Significant difference in age, BMI, duration of diabetes, economic development and the proportion of complications between the two groups are reached (Table 1).

**Table 1:** Clinical characteristics of T2DM outpatient participants prior to insulin therapy.

**Characteristics**	**Total (*n* = 49,119)**	**Basal insulin (*n* = 11,967)**	**Premixed insulin (*n* = 37,152)**	***P*-value**
Age, years	54.9 ± 11.4	56.3 ± 11.7	54.5 ± 11.3	< 0.001
Male gender	27,540 (56.1)	6,761 (56.5)	20,779 (55.9)	0.277
BMI, Kg/m^2^	24.2 ± 2.9	24.6 ± 3.1	24.1 ± 2.9	< 0.001
Duration of diabetes, year, median (IQR)	1.7 (0–5.0)	2.4 (0.25–6.1)	1.3 (0–4.4)	0.001
FPG, mmol/L	10.2 ± 2.8	10.1 ± 2.87	10.2 ± 2.79	0.001
< 7.0	2,621 (5.3)	599 (5.0)	2,022 (5.4)	0.064
≥7.0	46,498 (94.7)	11,368 (95.0)	35,130 (94.6)	
2h-PPG, mmol/L	14.9 ± 4.2	14.8 ± 4.1	14.9 ± 4.2	< 0.001
< 10.0	4,555 (9.3)	928 (7.8)	3,627 (9.8)	< 0.001
≥10.0	44,564 (90.7)	11,039 (92.2)	33,525 (90.2)	
HbA1c, %	9.0 ± 1.8	8.9 ± 1.8	9.0 ± 1.8	0.001
< 7.0	2,945 (6.0)	761 (6.4)	2,184 (5.9)	0.054
≥7.0	46,174 (94.0)	11,206 (93.6)	34,968 (94.1)	
Hospital level				0.940
Primary	1,113 (2.3)	276 (2.3)	837 (2.3)	
Secondary	10,365 (21.1)	2,521 (21.1)	7,844 (21.1)	
Tertiary	37,641 (76.6)	9,170 (76.6)	28,471 (76.6)	
Economic development				<0.001
Underdeveloped	9,939 (20.2)	1,709 (14.3)	8,230 (22.2)	
Intermediately developed	12,447 (25.4)	2,517 (21.0)	9,930 (26.7)	
Developed	26,733 (54.4)	7,741 (64.7)	18,992 (51.1)	
Complications and comorbidities	9,292 (18.9)	2,378 (19.9)	6,914 (18.6)	0.002
Hypertension	6,243 (12.7)	1,765 (14.7)	4,478 (12.1)	<0.001
Coronary heart disease	2,143 (4.4)	680 (5.7)	1,463 (3.9)	<0.001
Cerebrovascular disease	1,014 (2.1)	218 (1.8)	796 (2.1)	0.031
Dyslipidemia	3,860 (7.9)	1,104 (9.2)	3,756 (10.1)	0.004
Diabetic retinopathy	1,648 (3.4)	591 (4.8)	1,057 (2.8)	<0.001
Diabetic neuropathy	2,030 (4.1)	661 (5.5)	1,369 (3.7)	<0.001
Diabetic nephropathy	1,310 (2.7)	484 (4.0)	826 (2.2)	<0.001
Diabetic foot	204 (0.4)	75 (0.6)	129 (0.4)	<0.001

*Data was expressed as mean ± standard deviation or n (%), unless otherwise indicated. BMI, Body mass index; FPG, Fasting plasma glucose; 2h-PPG, 2-hr postprandial plasma glucose; HbA1c, Glycosylated Hemoglobin A-1c; IQR, interquartile range*.

### Comparison of basal and premixed insulin on HbA1c reaching goal

**Figure 1:**
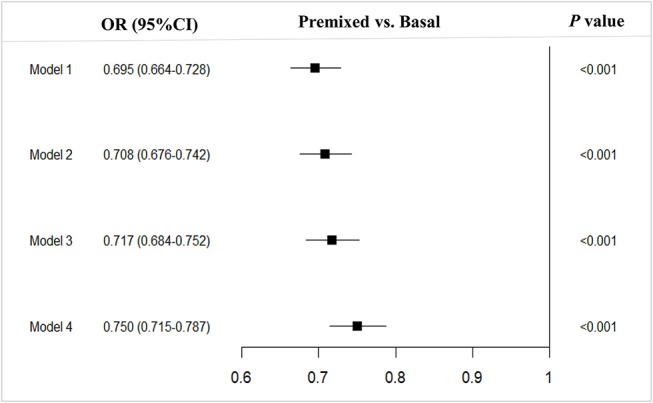
Multivariable analysis of type 2 diabetes outpatients on premixed and basal insulin treatments that reached the target HbA1c <7%. Model 1, adjusted for age prior to insulin therapy, sex, hospital level and economic development; Model 2, model 1 + adjusted for BMI and diabetic duration prior to insulin therapy; Model 3, model 2 + adjusted for FPG, 2h-PPGand HbA1c prior to initiating insulin therapy; Model 4, model 3 + adjusted for and complications. OR, Odds ratio; CI, Confidence interval; vs, versus.

Compared to the patients with basal insulin monotherapy, more patients with premixed insulin attained long-term glycemic control with a HbA1c < 7.0% (premixed with 33.1% (12278/37152) vs. basal with 25.5% (3058/11967), OR 0.695, 95%CI 0.664–0.728) in the univariate analysis. After adjusting for confounding factors (model 1 adjusted for age prior to insulin therapy, sex, hospital level, and economic development; model 2 adjusted for age prior to insulin therapy, sex, hospital level, economic development, BMI, and diabetic duration prior to insulin therapy; model 3 adjusted for age prior to insulin therapy, sex, hospital level, economic development, BMI, diabetic duration prior to insulin therapy, FPG, 2h-PPG, and HbA1c prior to insulin therapy; model 4 adjusted for age prior to insulin therapy, sex, hospital level, economic development, BMI, diabetic duration prior to insulin therapy, FPG, 2h-PPG, HbA1c prior to insulin therapy and complications and comorbidities prior to insulin therapy) in multiple logistic regression models, there were still more patients using premixed insulin attained long-term glycemic control of reaching the targeted HbA1c of <7% (model 1, OR 0.695, 95%CI 0.664–0.728; model 2, OR 0.708, 95%CI 0.676–0.742; model 3, OR 0.717, 95%CI 0.684–0.752; model 4, OR 0.750, 95%CI 0.715–0.787) than those using basal insulin monotherapy (Figure 1).

### Subgroup analysis of basal vs. premixed insulin on reaching target HbA1c < 7%

**Figure 2:**
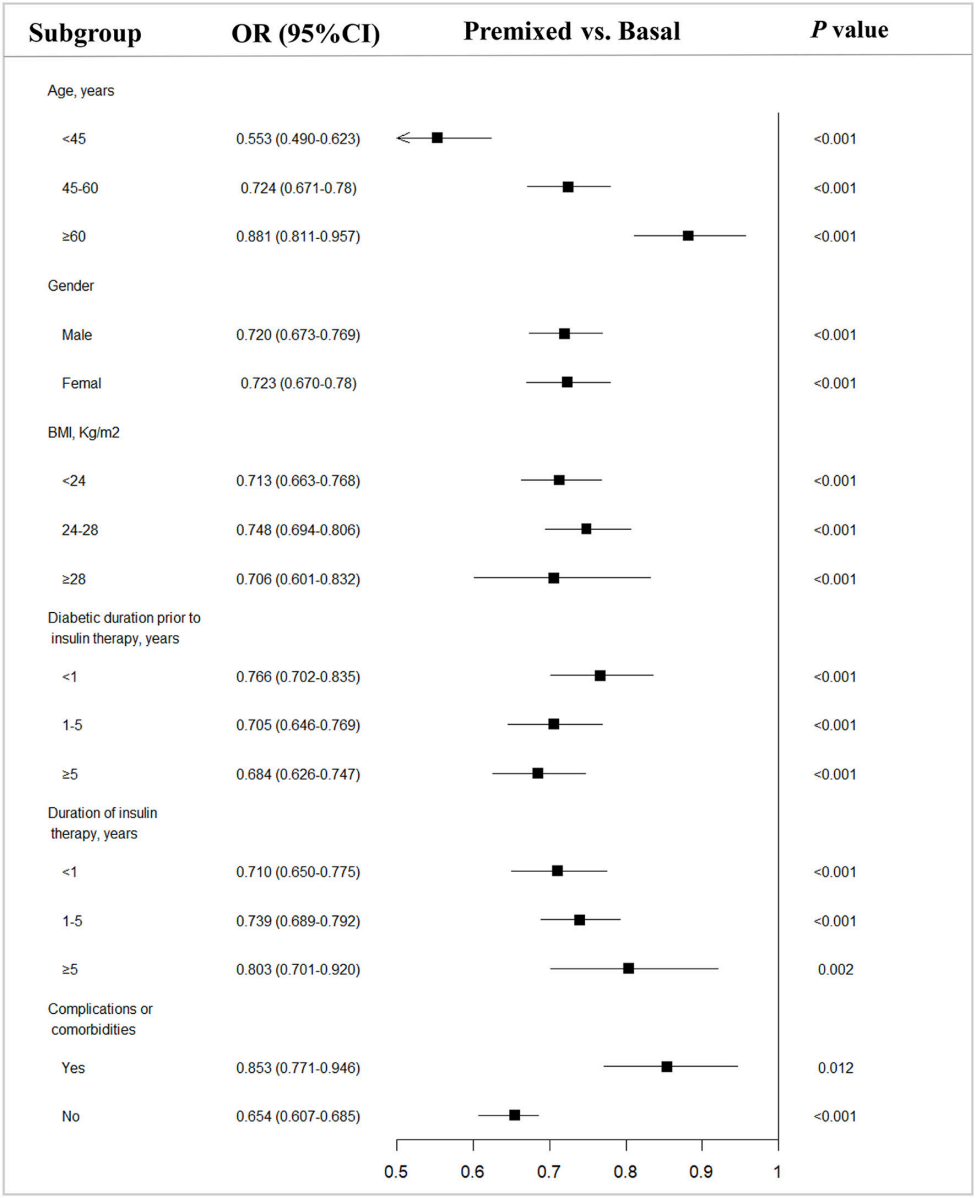
Subgroup analysis of type 2 diabetes outpatients on basal and premixed treatments reaching the target HbA1c <7%. OR, Odds ratio; CI, Confidence interval; vs, versus. *P*-value from subgroup analysis of multivariable logistic regression analysis adjusted for all factors.

Since there were difference in age, BMI, duration of diabetes, and the proportion of complications between the two groups prior to insulin therapy, to eliminate their influence on the efficacy of insulin therapy, we stratified the patients into subgroups by the potential influence factors, and subgroup analysis of these patients being able to reach glycemic control with target HbA1c of <7% were performed. The results showed that with each subgroup, there were more patients on premixed insulin monotherapy that reached the target HbA1c (HbA1c <7%) than basal insulin monotherapy in the univariate analysis (Table 2). Further, after adjusting the factors which might influent the effect of insulin treatment, the multiple logistic regression models in different subgroups analysis also demonstrated similar results (Figure 2).

**Table 2:** Insulin monotherapy in different subgroups of T2DM outpatients reaching the target HbA1c <7%.

**Characteristics**	**Total, *n* (%)**	**Basal, *n* (%)**	**Premixed, *n* (%)**	***P*-value**
**AGE GROUPS, YEARS**				
<45	3,742 (39.4)	570 (29.4)	3,172 (42.0)	<0.001
45–60	7,150 (30.5)	1,302 (23.8)	5,848 (32.6)	<0.001
≥60	4,444 (27.4)	1,186 (26.0)	3,258 (28.0)	0.013
**GENDER**				
Male	8,629 (31.3)	1,737 (25.7)	6,892 (33.2)	<0.001
Female	6,707 (31.1)	1,321 (25.4)	5,386 (32.9)	<0.001
**BMI GROUPS, Kg/m**^2^				
<24	7,861 (33.3)	1,428 (27.4)	6,433 (35.0)	<0.001
24–28	6,446 (30.4)	1,330 (25.1)	5,116 (32.2)	<0.001
≥28	1,029 (24.0)	300 (20.8)	729 (25.6)	0.001
**DIABETIC DURATION BEFORE INSULIN MONOTHERAPY, YEARS**				
<1	6,127 (30.2)	977 (24.5)	5,150 (31.6)	<0.001
1–5	5,196 (31.9)	1,051 (25.6)	4,145 (34.0)	<0.001
≥5	4,013 (32.0)	1,030 (26.6)	2,983 (34.4)	<0.001
**DURATION OF INSULIN MONOTHERAPY, YEARS**				
<1	4,920 (33.2)	1,148 (28.3)	3,772 (35.0)	<0.001
1–5	7,882 (30.2)	1,532 (23.8)	6,359 (32.2)	<0.001
≥5	2,534 (31.1)	387 (25.5)	2,147 (32.4)	<0.001
**COMPLICATIONS OR COMORBIDITIES**				
Yes	2,718 (29.3)	729 (30.7)	1,989 (28.8)	0.081
No	13,330 (33.5)	2,931 (30.5)	10,369 (34.3)	<0.001

*All P-value from Chi-square test*.

## Discussion

This large-scale survey demonstrated that T2DM outpatients treated with premixed insulin monotherapy had a better glycemic control reaching normal HbA1c of <7% than those outpatients on basal insulin monotherapy, and this result was further confirmed by the better efficacy of premixed insulin over basal insulin treatment stratified by the different subgroups of gender, BMI, duration of diabetes, presence of complications, and comorbidities. To the best of our knowledge, this study is the first to demonstrate this superior efficacy of premixed insulin over basal insulin treatment in attaining the target HbA1c for Chinses T2DM outpatients with insulin monotherapy.

Consistent with our results, the DURABLE study (20, 21) showed that a premixed insulin analog had a slightly higher proportion of patients attaining the target HbA1c than basal insulin. However, a systematic review recently reported that the change of HbA1c was not significantly different between the premixed insulin analog and basal groups (14). Several reasons might be account for the better efficacy of premixed insulin in our study. The premixed insulin contained the short or fast-acting insulin which was not included in basal insulin, whereby the short or fast-acting insulin would have more efficacious glucose-lowering activity for the postprandial rise in blood glucose (22). Since about half T2DM patients in China exhibited mainly an elevated postprandial blood glucose rather than basal blood glucose (23), this would partly explain the better efficacy of the premixed insulin treatment strategy. The mean duration in the insulin therapy we observed in this study was 2 years, whereas most clinical trials had a shorter observation duration, which could result in missing some patients who might have reached the target HbA1c.

The limitations of this study included: (1) we did not compare the adverse effect of basal and premixed insulin therapy, such as hypoglycemia events; (2) there might be some confounding effects from some factors that were not captured in this study that could have affected insulin actions, such a s genetic factor and behavioral factors, physical activity and diets; (3) probably because the endocrinologists are skilled at premixed insulin treatment for diabetes patients in participating hospital in China, the majority patients were assigned to the premixed insulin group, resulting in imbalance of patient's basis among both groups; and (4) the severe type 2 diabetes patients were likely to visit doctors, thus this study may include more T2DM patients treated with insulin combined with OADs or insulin alone, which leading a selection bias.

The strengths of this study included: (1) the large sample size which would reduce bias; (2) participants were a good representative cross-section of almost every region of China; (3) a relatively longer time of observation on the beneficial or lack of effect of insulin therapy than previous clinical trials; (4) the criteria for inclusion and exclusion were not as strict as the randomized controlled trials. The results from this study therefore more closely resemble the routine primary care of all T2DM outpatients who are being treated with basal and premixed insulin regimens, which should give the endocrinologists a better rationale in choosing the appropriate anti-diabetic treatment for their T2DM outpatients daily in China.

## Ethics statement

The study protocol was approved by the Ethics Committee of the Chinese People's Liberation Army General Hospital and informed consent was obtained from each subject before collecting data.

## Author contributions

GL carried out the statistical analyses and drafted the manuscript. YH reviewed and edited the manuscript. JD and LJ participated in the design and coordination of the study. YP, YY, and HZ had full access to all the data in the study and takes responsibility for the accuracy of the data analysis. HG contributed to correct English language. JL contributed to the discussion. All authors have modified and approved the final manuscript. YH is the guarantor of this work and, as such, had full access to all the data in the study and takes responsibility for the integrity of the data and the accuracy of the data analysis.

### Conflict of interest statement

The authors declare that the research was conducted in the absence of any commercial or financial relationships that could be construed as a potential conflict of interest.
